# Examining clinical leadership in Kenyan public hospitals through the distributed leadership lens

**DOI:** 10.1093/heapol/czx167

**Published:** 2018-07-08

**Authors:** Jacinta Nzinga, Gerry McGivern, Mike English

**Affiliations:** 1 Health Services and Research Group, Kenya Medical Research Institute/Wellcome Trust Research Programme, 197 Lenana Place, Nairobi, Kenya; 2 Warwick Business School, University of Warwick, Coventry, UK; 3 Nuffield Department of Medicine and Department of Paediatrics, University of Oxford, Oxford, UK

**Keywords:** Clinical leadership, distributed leadership, health care, LMICs, professionals, power

## Abstract

Clinical leadership is recognized as a crucial element in health system strengthening and health policy globally yet it has received relatively little attention in low and middle income countries (LMICs). Moreover, analyses of clinical leadership tend to focus on senior-level individual leaders, overlooking a wider constellation of middle-level leaders delivering health care in practice in a way affected by their health care context. Using the theoretical lens of ‘distributed leadership’, this article examines how middle-level leadership is practised and affected by context in Kenyan county hospitals, providing insights relevant to health care in other LMICs. The article is based on empirical qualitative case studies of clinical departmental leadership in two Kenyan public hospitals, drawing on data gathered through ethnographic observation, interviews and focus groups. We inductively and iteratively coded, analysed and theorized our findings. We found the distributed leadership lens useful for the purpose of analysing middle-level leadership in Kenyan hospitals, although clinical departmental leadership was understood locally in more individualized terms. Our distributed lens revealed medical and nursing leadership occurring in parallel and how only doctors in leadership roles were able to directly influence behaviour among their medical colleagues, using inter-personal skills, power and professional expertize. Finally, we found that Kenyan hospital contexts were characterized by cultures, norms and structures that constrained the way leadership was practiced. We make a theoretical contribution by demonstrating the utility of using distributed leadership as a lens for analysing leadership in LIMC health care contexts, revealing how context, power and inter-professional relationships moderate individual leaders’ ability to bring about change. Our findings, have important implications for how leadership is conceptualized and the way leadership development and training are provided in LMICs health systems.

**Key Messages**

- There is growing recognition of the importance of clinical leadership in improving health care services but in LMICs there is relatively little empirical research on the subject despite weak leadership and managerial capacities contributing to problems facing LMIC health systems.
- We use a distributed leadership lens to examine empirical data on clinical leadership in Kenyan district hospitals, providing contextually grounded lessons relevant to other LMIC health care settings, where clinical leadership remains underdeveloped.
- We found that medical professional dominance and parallel lines of leadership between nurses and doctors affected leadership in Kenyan district hospitals and undermined the development of a more distributed form of clinical leadership.
- Local cultures, norms and structures in health care organizations affect and constrain how leadership is practiced.
- Our analysis of clinical leaders at the middle of hospitals’ organizational hierarchy reinforces importance of clinical leadership at the operational level where key and critical service delivery decisions are made.

## Introduction

Leadership plays a key role in improving care quality, performance and outcomes in health systems globally (WHO, 2008; Gilson and Daire 2011; Alliance for health policy and systems, 2016) and having doctors and nurses in leadership roles has been found to be important in driving health service improvement (Ferlie and Shortell 2001; Ham, 2003; Fitzgerald *et al.* 2013; McGivern *et al.* 2015). However, there is relatively little empirical research on clinical leadership in low and middle income countries (LMICs) (van Lerberghe, 2008), despite weak leadership and managerial capacities contributing to problems facing health systems in these settings (Egger and Ollier 2007; Puoane *et al.* 2008; Marchal *et al.* 2010; Moyo *et al.* 2013).

Moreover, leadership in health systems improvement and strengthening is rarely discussed in a way informed either by leadership theory or an understanding of the ‘messy’ practice of leadership (Denis *et al.* 2010). Furthermore, leadership is usually conceptualized as a top-down and individualized phenomenon, including LMIC health systems. Yet health care delivery involves multiple actors (Denis *et al.* 2010), particularly powerful medical professionals (Freidson, 1970), who often make operational clinical decisions at ward level, in ways influenced more by collegial mechanisms than line management structures (Ham and Dickinson 2008). Accordingly, researchers have shown that leadership in health care usually involves multiple leaders from different professional groups, at the top and middle-levels of organizations, whose actions are enabled and constrained by their organizational contexts (Denis *et al.* 2001; Currie and Lockett 2011; Denis *et al.* 2012; Fulop and Mark 2013; Ferlie *et al.* 2013; Fitzgerald *et al.* 2013; Nzinga *et al.* 2013; Daire and Gilson 2014). Addressing this oversight, we the use lens of ‘distributed leadership’ (Gronn, 2002) to examine the messy day-to-day practice of middle-level leadership in Kenyan district hospitals.

District hospitals are an important part of health systems in LMICs, delivering essential health care services in resource poor settings (Hugo *et al.* 2010), although their functioning is not well understood (English *et al.* 2004; van Lerberghe, 2008). The limited literature on district hospitals in LMICs tend to focus on performance outcomes (Puoane *et al.* 2008; Hugo *et al.* 2010) and quality improvement in a decontextualized way (Elwyn *et al.* 2007). Yet hospitals are complex organizations, whose functioning and performance are determined by both formal and informal rules, regulations, cultures and norms (Kuhlmann *et al.* 2015). We focus on day-to-day leadership of middle level leaders during routine delivery of health care in Kenyan county (formerly district) hospitals.

The structure of the article is as follows. First, we outline theory underpinning our study and explain why distributed leadership is a useful lens for examining health care. We then describe the Kenyan county hospital context where our study was situated. We explain the methods we used to gather and analyse our qualitative data, before presenting our empirical findings and discussing their implication for health policy and practice.

### Distributed and socially constructed leadership

In health care, there is a complex inter-relationship between leadership, health professions, contexts and organizational performance (Ferlie and Shortell 2001; Goodall, 2011), so leadership cannot be conceptualized as a top-down and individualized construct. We therefore need a broader conceptualization of health care leadership, which encapsulates interactions between leaders, followers and contexts (Edmonstone, 2009; Chreim *et al.* 2010).

Distributed leadership therefore provides a useful framework for understanding how leaders and followers co-create a shared understanding of their daily interactions (Gronn, 2002; Spillane *et al.* 2004) in health care. Distributed leadership is defined as a constellation in which individual members play distinct roles and all members work together. It provides a holistic sense of leadership as a product of leaders and followers co-constructing performance in collective and group context, and provides a dynamic, non-linear frame on how people and events interact in organizations (Denis *et al.* 2001; Gronn, 2002). We use distributed leadership to frame the process of leadership as a co-construction of shared meaning and action to accomplish common objectives (Bolden, 2011).

Moreover, leadership includes a relational aspect involving power, relationships between actors involved and the context within which they operate. Thus, through social processes, such as building inter-personal relationships, influencing and motivating others, we shift from a perspective of ‘who is leading’ to ‘how leadership is created and accomplished’ (Uhl-Bien, 2006; Martin *et al.* 2009). Distributed leadership can also therefore be thought of a form of ‘relational leadership’; a process of social influence through which emergent coordination and change are constructed and produced (Uhl-Bien, 2006). Put simply, distributed leadership conceptualizes leadership as a collective practice embedded within a wider constellation of relations between leaders, followers and context (Gronn, 2002; Denis *et al.* 2012).

For Gronn (2002), there are two main dimensions of distributed leadership. *Concertive action* is about aligning the direction of leadership across different individuals, facilitating collaboration and sharing of leadership within work groups. *Conjoint agency* is about the nature and quality of interactions among leaders and followers; how leaders synchronize leadership acts through their individual plans, those of peers and a willingness to engage in mutual influence with one another (Gronn, 2002; Currie and Lockett 2011). Therefore, distributed leadership can be thought of as ‘a process involving multiple agents, including those who might enact leadership and those who might enact followership depending on context (Mehra *et al.* 2006; Gordon *et al.* 2015), involving the ‘influence-ship’ of both leaders on followers and followers on leaders. This reciprocal influence affects leadership actions whilst contingent on the context in which the interactions happen.

Context, including organizational structures, routines, socio-cultural, political and historical elements, is an important element in the conceptualization of the dynamics between leadership and followership (Spillane *et al.* 2004). Context enables and constrains leadership practice and, as such, leadership can be thought of as an emergent, on-going negotiation between social actors in co-constructing meaning, trust and cohesion and better practice (Bolden, 2011).

While there has been increasing use of distributed leadership as theoretical ‘unit of analysis’ (Gronn, 2002) in analysing health care leadership particularly in HIC settings (Currie and Lockett 2011; Fitzgerald *et al.* 2013; Ferlie *et al.* 2013), distributed leadership has not been applied in LMICs. Yet using the distributed leadership lens is critical in LMIC health system contexts, because, in the frequent absence of effective standardized processes and accountability mechanisms, its governance is affected by plural and contextually situated modes of professional organization. Thus, we use the distributed leadership lens to examine clinical leadership in Kenyan county hospitals, which are similarly embedded in wider complex healthcare contexts. By focusing on county hospitals in one LMIC, we show how distributed leadership provides a useful lens for understanding clinical leadership and, in doing so, provide lessons for others analysing leadership in other LMIC health care contexts.

### The Kenyan health care context

In Kenya, county hospitals serve critical roles as the first level of referral care, while also providing support to peripheral health facilities such as health centres, dispensaries and the community. Training of physicians, clinical officers, nurses and on-going medical education are all provided by the county hospitals. County hospitals consume about 50% of all funding allocated to the Kenyan health sector (Mills, 1990; Barasa *et al.* 2015) and employ half of all public health care staff. Improving the way Kenyan district hospitals are led and managed could therefore have a significant impact on the country’s health system. Unfortunately, the performance and quality of Kenyan public sector hospitals are often poor (English *et al.* 2004; Irimu *et al.* 2012) due to resource and structural limitations, inadequate leadership and poor communication between senior and frontline workers (Nzinga *et al.* 2009; English, 2013).

**Figure 1.**
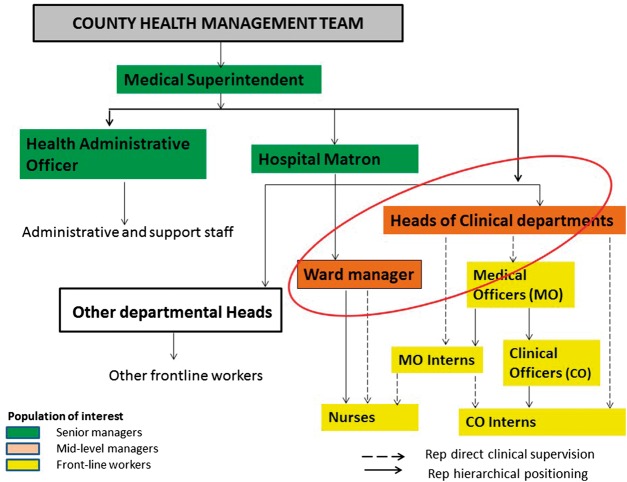
Generic organogram of county hospitals in Kenya with the circle representing the mid-level leaders of interest for this study

County hospital heads of departments, including those clinically and non-clinically trained, form the middle level leadership of these hospitals and play a key role in making improvements in Kenya county hospitals. Our focus is on these middle level leaders running clinical departments and supervising front-line workers (principally doctors and nurses) (Nzinga *et al.* 2009; Nzinga *et al.* 2013). All middle level leaders report to a senior leadership team, comprising a medical superintendent (a doctor) and a hospital matron (the head of nursing), supported by a health administrative officer (without clinical training) (see Figure 1 below), who are in charge of translating health policies into practice. Senior district hospital leaders may also have regulatory roles at county and national levels (English *et al.* 2004).

Clinical departments in Kenyan district hospitals (e.g. medicine, paediatrics, obstetrics and gynaecology and surgery) are jointly managed by doctors and nurses (see Figure 1 above). Doctors heading these departments may have a higher degree in an appropriate specialty or, especially in smaller rural hospitals, a general medical qualification. Nurses ‘in charge’ of inpatient wards and outpatient departments tend to have more work experience than junior doctors, although few have higher training in a specific clinical specialty (Nzinga *et al.* 2013). Senior managers and frontline workers alike expect doctors running departments to implement policy, lead and motivate staff to improved service delivery, despite few such doctors having leadership or management training (Nzinga *et al.* 2009; English *et al.* 2011).

The poor performance of hospitals in Kenya and other LMICs is often attributed to poor leadership at operational level (Nzinga *et al.* 2009; English *et al.* 2011), yet such leadership is often situated in a complex healthcare context that undermines leaders’ abilities to act. For example, decentralization of governance of health services in Kenya and increasing accountability demands on clinicians taking on leadership and managerial roles (KPMG, 2013) make the enactment of leadership roles difficult. Consequently, our research question is: ‘how are leadership micro-practices at the middle level of hospitals (clinical departments) negotiated and enacted?’

## Methodology

This article is based upon qualitative case studies of two Kenyan public county (district) hospitals, focusing on eight mid-level departmental leaders (four in each hospital) running front-line clinical departments [four medical consultants (three male and one female) and four nurses ‘in charge’ of inpatient wards (all female)]. Between February and September 2014, the lead author spent 480 h shadowing and observing these leaders’ routine hospital work, including during clinical ward rounds, departmental meetings, hospital management meetings and continuous learning (continuing professional development) sessions running clinics run (*see interview and observation guides in*Supplementary appendix).

The lead author interviewed each of the clinical departmental leaders three times, asking questions about what influenced them to pursue clinical training, how they came to be appointed as heads of departments, day-to day leadership in terms of how they interpreted behaviours acts and experiences, their roles and achievements as departmental leaders. She also interviewed 3 senior managers, 4 mid-level leaders and 21 frontline workers in Hospital A and 3 senior managers, 4 mid-level leaders and 16 frontline workers in Hospital B during one-to-one interviews and focus groups. She asked questions about perceptions of leadership in the departments run by the eight departmental leaders. Thus, in total, 61 people were interviewed across the two hospitals.

We managed and coded data using NVIVO 10 Qualitative data software. We then theorised data drawing on Gioia *et al.* (2013) inductive and Corbin and Strauss’ (2008) grounded research methods. We started with open coding, looking for inductive concepts and themes (also informed by relevant literature), then axially coded these data, allowing concepts to emerge, while developing relationships and patterns among categories and themes. We then compared concepts emerging from data with leadership literature, taking an iterative approach to theorization (Eisenhardt, 1989; Golden-Biddle and Locke 2007) to explain the social mechanisms and processes through which leadership is enacted in the empirical sites we studied.

## Results

We now describe and explain our empirical findings.

### Perceptions of leadership as an individualized phenomenon

While we used a distributed leadership lens to analyse mid-level leadership, interviewees perceived leadership as individualized, top-down phenomenon, in which clinical departmental heads were expected to tell clinical staff what to do. As a result, followers demonstrated little personal agency. As a consultant paediatrican leading a department noted:

*‘When I left, some of my staff felt lost because I was not there to give them direction … I felt like I had not build structures to support things. I felt like I was the one man show but I said that has to change … they should not think that I should always be there for things to go on.’* ***(*** ***Paediatric consultant*, *Hospital A*** ***).***

Most respondents also conflated leadership with being a departmental ‘figure head’, ‘spokesperson’ and ‘role model’, as noted below:

*‘Our consultant is hilarious and so, so good. He knows his stuff also and … is not [just] … focused on medicine and the patient … he brings some social aspects, cultural aspect.’ (* ***Medical officer intern, obstetrics/gynaecology rotation, Hospital B*** ***).***

*‘They expect you to be the role model in everything, even just coming on duty, putting on proper uniform, even the language. Even in the working … they expect you to show them. You teach them OK, mostly they always act like we do.’ (* ***Nurse manager, Maternity ward*, *Hospital A*** ***).***

Heads of departments’ formal responsibilities and accountability within the departments under-pinned the individualized view of leadership. As a medical consultant running a department noted:

*‘My role as head of the department is to make sure that everything in the pediatric department is running. Doing daily ward rounds, outpatient clinics and specialist clinics … academic mentorship to clinical officers to medical officers and interns.’* ***(*** ***Pediatric consultant, Hospital B*** ***).***

### Leadership along professional hierarchies

A key feature of the context in which middle-level leadership occurred in district Kenyan hospitals was inter-professional stratification, particularly between doctors and nurses, producing parallel lines of leadership. Nurse ‘in charges’ supervised nurses in departments, whose work plans were developed separately from those of medical officers, medical and clinical officer (non-physician clinicians) interns, who were supervised by medical consultants, as described below:

*‘When it comes to the CO [clinical officer] interns, there’s a bit of interference from their in-charge. For example, you might have a number of CO interns in your rotation, and then you come on a random day and you find the CO in-charge has actually deployed them somewhere else to do some work, and a Head of Department, you really have no powers to contest that. The nurses, we have always worked as parallel systems, so the nurses have their own way of reporting and the Medical Officers also have their own way of reporting but we’ve never had that clash, somehow we’ve been able to accommodate each other. But that doesn’t seem to happen with the CO interns because there will be some decision from their in-charge and somehow that decision will be … there’ll be very little that you can do to influence that decision when it’s made. So yeah, that again is quite a challenge I would say from the admin side.’ (****Obstetrics/gynaecology consultant, Hospital B).***

Relationships in clinical department also developed around professional specializations, with limited opportunities for different professional groups to meet and discuss departmental issues as a team. From observations, meetings were cadre specific and nurses and doctors rarely interacted. Even where standard operating procedures were designed to be multi-disciplinary, they were not always enacted in multi-disciplinary ways, as the following interview extract indicates:

*‘The collaboration between us and nurses … could be better. For example, when we hold mortality meetings, the nurses should be there but often … they are not and also we rarely see them (nurses) join ward rounds.’****(Paediatric medical officer, Hospital A).***

*‘We even have Continuous Medical Education (CME) every 2 weeks but we can’t attend, we have so much work, so you don’t really have time for CME’s.’****(Paediatric nurse, Hospital A).***

Doctors usually made departmental decisions individually, without involving their teams or nurse managers within the same department. Nurses also made decisions on ward operations independently, without involving their nursing teams or medical consultants.

Despite hospital administrators recognizing problems resulting from parallel lines of leadership, it was accepted as a cultural norm and remained unaddressed, undermining the possibility of team or distributed leadership, as indicated by the interview extract below:

*‘Well we have work plans per departments and the nursing staff, they do their work with the nursing manager based on their profession. The doctors will do their work with their consultant in their department but the only challenge that we have had is marrying the work plan of the nurses and that of the clinicians. So that gap is there and we are still thinking of another way to address this.’ (****Medical Superintendent, Hospital A).***

Respondents described medical dominance within the inter-professional hierarchy affecting leadership in these hospitals. As a medical head of department noted:

‘As consultants, we are the top leadership of the department, so we make the decisions on everything.’ **(Obstetrics and Gynaecology Consultant, Hospital A).**

Clinical heads of departments’ senior medical professional identity, presumed clinical knowledge and expertize appeared to provide taken-for-granted authority in leadership roles. For example, a medical officer described the consultant leading their department as:

*‘Someone who wasn’t just given a head of department position, that it is someone who is very knowledgeable.’****(Medical Officer Intern***, ***Obstetrics and Gynaecology, Hospital B).***

Our observations suggested that even inexperienced medical doctors had authority over nurses. So, nurse managers with more technical experience struggled to exercise authority over the medical interns. A nurse noted:

*‘When the clinical interns come, they look down upon you. But you see, I’ve worked in paediatrics for long, so I know what the consultant expects. So, when you are trying to tell that intern, he’s like ‘who are you?’ (****Paediatric Nurse Manager, Hospital B).***

Nurses’ experiential knowledge was also less valued within the clinical departments and nurse leaders were expected to play supportive roles to doctors. As a consultant noted:

*‘We (medical doctors) are the main decision-makers in the ward … but for the supplies and resources generally … you have an efficient nurse who makes sure all of that is delivered.’****(Paediatrics consultant, Hospital B).***

Few nurse managers appeared empowered by their leadership role. For example, even a nurse in-charge of paediatrics, who interviewees considered charismatic, motivating and inspiring did not consider herself a leader. As she commented:

*‘I am someone who minds my own business and I don’t see it as a short coming and I like seeing things organized … that is just my initiative … another person without that character … will do the bare necessity.’ (****Nurse Manager, Hospital A).***

Nurse managers often appeared approachable, empathetic and understanding towards team members, using informal inter-personal relationships to influence change, as the following interview extracts suggest:

*‘[Nurse manager] really tries his best to balance being an administrator, a teacher and also a friend. He tries to know what’s going on in people’s lives, so he tries to reach out and he is outgoing … he is very good with the nurses.’****(Maternal and child health nurse, Hospital A).***

*‘As a departmental head … first of all you listen to them [nurses] and understand that each one of us has got problems and you are dealing with adults … if you don’t solve their problem, then you are even creating problems for yourself.’****(Maternity nurse Manager, Hospital B).***

During observations of hospital management team meetings nurse managers played silent and supportive roles, unable to challenge the perceived expertize and authority of medical professional colleagues. A nurse reported:

*‘Our nurse manager is supportive, a team player but with the hospital administration he feels intimidated. He cannot report to the administration the needs of the department because he is afraid that he may be pinned down there, so when he comes back to us, he will just be silent.’ (****Paediatric nurse, Hospital B).***

Only a few clinical departmental leaders, particularly those with social skills and knowledge of the local hospital context, had the authority and credibility to actively solve problems, as a consultant explained:

*‘[I] solve problems rather than blaming others or shifting problems to others. Like if there is no oxygen for patients who need it, I won’t start saying that the administration is not giving them oxygen, I will look, talk to the maintenance; ‘what is your problem?’ Maintenance will tell me it is procurement. Procurement will tell me we have a debt. So, I know the whole side of things. I actually went to see what the problem is, so I think that is what has helped me.’****(Paediatric consultant*, *Hospital A).***

More commonly, however, we observed medical consultants using coercive power and intimidating junior staff to make things happen, as a medical officer describes below:

*‘The way she [departmental leader] talked to us! She would tell us sometimes: ‘I don’t trust your decisions; see the way you make poor decisions’ … all those bad things. She was not encouraging, she was finding fault at your decisions, and doing it in front of the patients. She was not encouraging.’****(Medical officer intern, Paediatrics rotation, Hospital B).***

Clinical departmental leaders rarely recognized effort or praised their teams and were more likely to point out inadequacies and failures. This created a blame culture and poor inter-personal relationships, which subsequently became accepted as the norm. Another medical officer intern noted:

*‘Nobody will applaud you for the good things, the bad things will be detected.’ (****Medical officer intern, Paediatrics rotation, Hospital B).***

Intimidation was also seen to characterize senior management:

*‘[Senior managers] play the intimidation game. They tell you, if you do this we will not pay you.’****(Medical officer, Paediatric rotation*, *Hospital A).***

Top-down communication was seen to be problematic too:

*‘As a team leader, communication downwards or upwards it is a challenge … communication from the topmost administration … is tricky.’ (****Nurse ‘in charge’, Paediatrics ward, Hospital B).***

In sum, inter-professional hierarchies and boundaries significantly affected mid-level leadership practices, with doctors ‘naturally’ assuming leadership roles, due to their perceived credibility and expert medical knowledge, while nurse leaders played quieter supportive roles.

### How context shapes and is shaped by leadership

Interestingly, we found little difference between patterns of leadership in the two hospital we studied. In both hospitals, departments usually lacked standardized ways of working, clear goals, aims, job descriptions, accountability and supervision. Without these procedures, mid-level leaders were, in effect, often unaccountable for their own and their teams’ conduct. Simultaneously, inertia was deeply embedded within the hospital cultures, meaning that clinical staff simply ignored problems, as described below:

*‘There are conflicts or disagreements in this ward … We don’t bring it up. You keep quiet and it goes away … The victimization is really a lot in this hospital. You don’t go and report because if you do it will come back to you.’****(Medical officer, Gynaecology*, *Hospital A).***

We also observed the way conflicts, poor practices, negative work climates and health worker norms were both accepted and taken-for-granted and leaders’ ignorance (or ignoring) of such issues only reinforced this. Thus, negligent practices, even those resulting in fatalities, simply went unreported, as the interview extract below describes:

*‘You have called the anaesthetist at 2 p.m., the guy shows up at 6 p.m. You go in and remove the dead baby, who was alive from 2 p.m. to 5 p.m., and you are removing the foetus at around 5.30–6 p.m. I am afraid of going to report this guy, because it will come back to me and they will say I am the one who reported him. So, you just keep quiet and maybe when the case is taken upstairs and when the matron looks at the file then she will summon him.’****(Medical Officer, Obstetrics and Gynaecology, Hospital A).***

While nurse managers were continuously present in the hospitals, clinical consultants were often absent, some spending only a few hours in the public county hospitals per week. However, the few middle-level medical leaders who were physically present in their clinical departments had made significant effort and progress in improving service delivery. For instance, one ward, which stood out in terms of cleanliness, staff punctuality and high quality, team-based patient care, was led by a consultant paediatrician who, from our observations, role-modelled good clinical practice, inter-personal relationships and behavior expected of staff. The consultant noted:

*‘You can drive the agenda … people used to start ward rounds at 9 a.m. … continue to 1 p.m. visiting hours. But now we have been starting our rounds at 8 a.m. And we have been having a feedback-like report in the morning. So that the person on night duty tells us what happened at night. As a head of department actually you … can bring in such changes.’****(Paediatrics Consultant, Hospital A).***

So, while ineffective managerial procedures, inert organizational culture and poor practices were accepted as the norm, where doctors in leadership roles were motivated to do so, they could bring about improvements to health care delivery.

## Discussion

Using distributed leadership as the unit of analysis (Gronn, 2002), we examined leadership in Kenyan hospital departments at micro-level, focusing on individual leaders (clinical heads of department and nurse ‘in charges’) situated within organizational context and social processes, involving interactions between multiple professional actors. Four key themes emerged from our analysis.

First, we found clinical departmental leadership was heavily affected by taken-for-granted individualized concepts of leadership, top-down authority and medical professional dominance, reflecting other research on leadership in Kenyan health care (Nzinga *et al.* 2009), other LMIC health care systems and global health care more generally (Freidson, 1970; Denis *et al.* 2001; Ferlie *et al.* 2013). Thus, leadership in such settings cannot be explained in individual terms but ought to be considered in relation to organizational structures and wider (inter)professional norms.

Second, our research shows how power is fully implicated in leadership, reflecting existing research (Smircich and Morgan 1982; Pfeffer, 2010). Indeed, Kenyan hospital managers have been shown to be powerful actors expressing ‘power over, power with, power to and power within’ (VeneKlasen *et al.* 2002) routine hospital priority setting activities (Barasa *et al.* 2016). Likewise, we found that professional ‘expert power’ (Raven, 1992) to be a crucial component of leadership in LMIC healthcare, anchored particularly in clinicians’ specialized knowledge, which was often uncontested in Kenyan hospitals. Indeed, most mid-level leaders in our study relied on their expert power to lead departments and influence colleagues and juniors. Moreover, because of their dominance within the professional hierarchy, and greater representation in hospital management meetings, doctors were able enact leadership roles in the Kenyan county hospitals in ways that could potentially influence how health care was delivered. Such professional power is so deeply embedded and taken for granted in health care, that the associated problems it also propagates appearto be accepted. Thus, professional power and politics may also undermine the development of distributed leadership, where it requires power to be exercised at all organizational levels and by different professional cadres (Gordon *et al.* 2015).

Third, leader-follower relations occurred along cadre-specific lines, affected by professional power and social identities, with little multi-disciplinary interactions or conjoint agency (Gronn, 2002). Within their profession, medical consultants and nurse leaders were seen as knowledgeable experts, expected to provide coaching and mentorship to junior professional colleagues. Yet there was little inter-professional collaboration, multi-professional teamwork or diffusion of knowledge and experience across professional cadres, which distributed leadership requires. This may require leadership building trust, respect and inspiring common goals across professions (Mehra *et al.* 2006).

An emerging and related observation is that hospital leaders require leadership training and development to understand and address the contextual, (inter)professional and political factors affecting their ability to change and improve health care systems. Such software skills, including understanding how to use different sources of power, engage in local politics and cultivate facilitative relationships, are vital leadership skills.

Finally, we found a general pattern of inertia in the hospitals we studied. However, mid-level leaders with intimate knowledge of their organizations and informal social networks can negotiate and influence change in ways that senior leaders cannot (Huy, 2001; Dopson and Fitzgerald, 2006). Moreover, middle level leaders spend significant amounts of time communicating information, providing a useful resource in connecting with others (Nzinga *et al.* 2013) and developing shared meanings (Rouleau and Balogun 2011). However, in our study poor communication structures between senior and middle-level leaders and between mid-level leaders and their teams resulted in individualized, professionally dominated models of leadership, which often perpetuated apathy and inertia among followers. Yet, in rare cases, departmental medical leaders, who were physically present in their hospital departments, motivated improved work practices, role-modelled good professional practice and behaviours, and developed inter-personal and inter-professional team work, did make some changes.

### Implications for policy and practice and future research

Our study has implications for health care policy and practice in Kenya and other LMIC contexts. Firstly, our findings highlight the critical importance of reconceptualizing leadership in distributed rather than individual terms; as a collective social process situated in context and affected by (inter)professional politics. Second, leadership training accordingly needs to focus on developing conceptual, analytical and political skills to resolve the complex problems leaders face in practice, rather than concentrating only on technical skills and competencies, as is currently the case in Kenya and other LMICs. Such training needs to be contextually rich, to help leaders diagnose organizational contexts, understand the political consequences of their actions, particularly for professional hierarchies, to develop relationships and learn to use power to bring about constructive and sustainable change.

Moreover, where effective hospital departmental leaders are spotted, they need to be nurtured and brought together with other like-minded and talented leaders (Lehmann and Gilson 2013; Lehmann and Gilson 2015). Leadership that ignore contexts, professional authority, relations and power will do little in strengthening health systems and remedying the many significant problems facing health care systems in LMICs.

Future research might attempt to explore the development and implementation of leadership training programmes providing contextually embedded software skills and test their impact on leadership and hospital performance.

## Conclusion

This article explains mid-level leadership on the front line of health services in Kenyan district hospitals from a distributed perspective. It provides contextually situated lessons for those seeking to understand and develop leadership in other LMIC health care settings, where such research remains underdeveloped. Indeed, to the best of our knowledge, our study is the one of the first using the distributed leadership lens to understand healthcare leadership in LMICs.

We argued that using a distributed leadership lens to analyse leadership in LMIC health care, rather than individual ‘leader’ oriented perspectives, is crucial because of (inter)professional power, politics and parallel leadership between nurses and doctors. Indeed, these are also likely to undermine the development of distributed modes of leadership in practice. By focusing on everyday leadership practices, we provide descriptions of complex and relational distributed leadership processes in which the exercise of power is critical to influencing change. Our findings have implications for health leadership and managerial development programmes, which tend to focus on technical skills but ignore software skills and the way power, politics and context influence leadership practices and outcomes.

## Supplementary Material

Supplementary Data
