# Supplementary material for: Examining clinical leadership in Kenyan public hospitals through the distributed leadership lens
Source: Health Policy Plan. 2018 Jul 8;33(Suppl 2):ii27–34. doi: 10.1093/heapol/czx167 (PMC6037084; doi:10.1093/heapol/czx167)
Supplement: Supplementary Data [file czx167_suppl_data.pdf]

Mid level Managers in Kenyan County Hospitals: Tools

Tool A: Senior Managers Key informant Interview Guide

Aim: To understand how MLMs are selected and appointed, how senior level managers perceive MLMs roles and role enactment and to describe the kind of capacity and support that the MLMs have and/or receive for management practice

1. Please describe who the heads of department are in this facility?
    - Clinical side of things
    - Nursing side of things
2. How do they become HODs
    - Clinical consultants
    - Nurses in charge of the wards
3. What do actually they do?
    - As part of routine hospital activity?
    - Over and above routine work
    - Is there any guidance from the MOH on what they ought to be doing?
4. What is expected of the HODs?
    - By the senior management?
    - By peers?
    - By juniors
5. How much do the HODs influence in the hospital?
    - How much power is delegated to them?
    - How is their performance evaluated? By whom?
6. What are the goals of the hospital?
    - Are the HODs involved in their implementation? How?
7. How are they supported?

Tool B: MLM Narrative Interview Guide

Aim: To collect life experiences of MLMs from early career entry to progression as managers so as to tell a sequenced story of the MLMs journey into management with a goal of understanding how MLMs identify with managerial responsibilities and their feelings towards acceptance and enactment of this role.

1. What can you tell me about yourself?
   - Basic demographic information
   - How did you become a nurse/doctor? What was appealing about health care?

   Tell us how you became a nurse/a paediatrician / senior nurse?
   - What was appealing about this?
   - Could you share with us some of the life experiences that contributed to you choosing this path?
2. What do you do on a daily basis here at this hospital?
   - What do you spend most of your time doing?
   - What do you think are the most important roles? Why?
   - What do you least like doing? Why?
3. How long have you been 'in-charge' as a paediatrician / senior nurse? (Here? Elsewhere?)
   - What do you feel you are you in charge of? What do you feel you should be in charge of?
   - Is the role as you imagined it or is it different (in what ways)?
   - Is being a paediatrician / senior nurse something you knew much about before you took the task on?
4. Did anyone guide you on what was expected of you?
      - Formal JD;
      - Informal
   - Is there any real discussion about what the job is amongst colleagues and seniors?
   - What type of expectations are there of your profession?
      - Can you give any examples of how you have 'learned' what to do in your role (or from whom)
5. Do you have a team you work with? Who is in this team?
   - Are they aware of their roles?
      - Do you think it's important that they do? Why?
      - Do you have a role in clarifying their roles?

Tool C: MLM Key Informant Interview Guide

Aim: The purpose for this kind of interviews is to determine specific competencies and how they are deployed by MLMs in their' day to day life events, how they approach the management job, how they practice management practices and interpretation of day to day experiences.

1. How do you characterize your experience as a HOD?
    - What do you enjoy about your work?
    - What energizes you to keep going in your job?
2. As a manager/leader, do you have goals?
    - How do you evaluate your success at reaching those goals?
    - Do you have any values that guide your work?
    - Why are these goals and values influential in your job?
3. What do you aim to achieve in this department? Do you have a mission/vision statement for your department?
    - How was it created? What does it mean?
    - How do you communicate it to the rest of your team?
    - How do you ensure your team is motivated towards that same mission/vision?
    - What things have you tried to ensure that your team is working well?
    -
4. In your work, do you delegate your responsibility?
    - Can you give examples? How much? How often?
    - What criteria do you use to select whom to delegate to?
    - How do you check that the task given is done?
    - What are the common outcomes? In case of failure what happens?
5. In your work experience, are hospital goals/policies communicated for your department?
    - Has there been a new policy introduced in the hospital?
        - How was it done?
        - How were you involved?
    - How often are institutional routines broken (examples)? In such instances, what guides you on what action to take?
6. Have you experienced conflicts in your department? (Give recent examples)
    - How are conflicts resolved in your department? How?
    - Have you experienced inter-cadre conflicts and how do you resolve them? How?
7. Do your team members come to you with personal concerns?

- How do you handle such?
- Who do you go to with personal concerns? Why?

8. How do you value your team member's efforts?
    - Why?
    - Can you give examples?
- Do you interact with your team members outside the work setting?
- Do you use this interaction to strategize about the department's operations?

Tool D: MLM Reflective Interview Guide

Aim: The aim of these interviews is to explore meanings of the managers' life events and work experiences as perceived by the managers. Additionally this interview will help MLMs make meaning of their experiences, values, knowledge and skills as a basis for improving their management and leadership practices. For the researcher it will provide space opportunity to retell and interpret the facility managers' stories as precisely as possible.

1. Is being a HOD a role you have come to like?
    - Why / why not?
    - Do you feel engaged in this role? Why?
    - Do you think your team works well? Why?

2. Have you found this role to be rewarding?
    - What purposes has it served?
    - What has it enabled you to do that you value?
    - What did you have to do to achieve these things

3. Do you think you have come to be good at it?
    - Can you give some Examples?
    - Any examples that did not go so well?
    - What have you no control over?

4. What are the main challenges (probe beyond resources to senior admin, team, people and what the role demands of them are beyond their technical knowledge)?
    - Is there anything about this role that frustrates you? Why?
    - If you were no longer a departmental head, is there anything you feel that you might miss?
    - What do you think/feel you can do to improve things? Why?

5. How would you like your work to develop in the future?
    - What are the positive things in your work environment that can help you get there?
    - What are some of the challenges you may face in getting there?

6. Has being a departmental head affected your hopes, dreams, aspirations and direction in life?
    - What would you say to more junior people in your profession or thinking of entering your profession about the role?

Tool E: Front line workers Interview Guide

Aim: To give voice to the informant's of the story by allowing the front line workers to narrate how leadership and management are enacted in their daily routines by the MLMs, to understand the identity of the MLMs from the perception of their juniors and document the dynamic relationship of MLMs and their followers.

DOMAIN	SAMPLE QUESTIONS
**Demographics**	Please tell us;<br>• What is your educational background post secondary school?<br>• How long you have worked in this hospital?
**Communication**	What is your job description?<br>• How were these communicated to you?<br>• Do they match the tasks actually undertaken?<br>What are the min things that this department is trying to achieve?<br>• Do you know what the goals of this department/ward are?<br>• How did you learn this? What does it mean to you?<br>• Is it meaningful to you?<br>Are there any meetings or forums for you to learn this?

	• With whom?<br>• How frequent?<br>• In what form?
**Strategist and negotiator**	Do you think the hospital has a clear idea of how it wants to develop?<br>• How did you come to learn this?<br>• Are staffs involved in efforts to make these things happen? How? Examples?<br>Can you describe your team's challenges? Other than supplies and staff shortages?<br>What does your HOD do to remove some of these challenges?<br>• Give two recent examples?
**Mentor and Coach**	Who helps you develop your work and enhance your skills?<br>• How do they do this?<br>How are performance evaluations done?<br>• Who does them?<br>• How frequent?<br>• Does he/she give you feedback?<br>• Is it useful?
**Decision making and problem solving**	Are you involved in decision making about the ward/department issues?<br>• Give a recent example<br>Are other departments that work closely with yours involved in decision making?<br>• Give a recent example<br>How are conflicts resolved in your department when they arise?<br>• Give a recent example
**Delegation and Accountability**	How does your HOD share work amongst everyone in the team?<br>• What criteria do they follow? Is it fair?<br>• Is it clear who is being given responsibility? Are they given any help?<br>• How are their efforts checked when tasks are delegated?<br>• What are the common outcomes in such occasions?<br>In case of failure in situations where the HOD had delegated to your team, what course of action is taken?
**Respect and Trust**	Do you feel your HOD represents you?<br>• Do you think they have your/team members' interests at heart?

	• Do you feel you can take your s personal concerns and work problems to your HOD?<br>What principles/values are you expected to work by in this department?<br>• How does your HOD make sure that you do this?<br>Is your HOD liked inside and outside the team?<br>• Why? Examples?
**Therapist and Motivator**	Are you valued in this department? In this hospital?<br>• How?<br>• Do you feel that your opinions and concerns are listened to?<br>How would you describe your relationship with your HOD outside the work environment?<br>During these interactions, do you discuss implications of ward/department operations
Any Questions for me?	

Tool F: Non participatory Observation Guide

Aim: In an informal manner this method will include watching as well as listening of the activities listed below (guide). While non participatory in nature, the observations will involve probing what is being observed from the facility managers as well as the clinic staff. The observations will allow the researcher to familiarize with the facility managers working context and to engage with the participants to build rapport, to break down distinctions between the researcher and participants, and to have an insider perspective especially in terms of physical availability of leaders

TYPE OF ACTIVITY TO BE OBSERVED	SOURCE OF OBSERVATION
Day to day clinical management of patients	Ward rounds and clinics
Mentoring and coaching of staff	Ward rounds, clinics and CMEs
Supervision of staff	Ward rounds, clinics and informal chats& encounters in the clinics and in the wards
Meetings with HMT	HMT meetings, informal chats& encounters

Meetings with staff	Staff meetings (may be cadre specific), informal chats& encounters
Interaction with senior staff	informal chats& encounters
Interaction with peers	Ward rounds, clinics informal chats& encounters
Interaction with juniors	Ward rounds, clinics informal chats& encounters
Other Admin duties	informal chats& encounters in clinic and in the wards

Other than the above, I will be walking around the hospital and talking to some of the key staff with an aim of observing and documenting the following

- Description of the facility
  - ✓ Organogram
  - ✓ Volume of patients
  - ✓ Organizational changes post devolution
- Organization of services
  - ✓ Lay out of service points
  - ✓ Patient flow
- Human Resource Capacity
  - ✓ Number of staff
  - ✓ Staff mix
